# Pediatric Point-of-Care Lung Ultrasonography: A Narrative Review

**DOI:** 10.5811/westjem.2022.3.54663

**Published:** 2022-06-05

**Authors:** Munaza Batool Rizvi, Joni E. Rabiner

**Affiliations:** Columbia University, Vagelos College of Physicians and Surgeons, Division of Pediatric Emergency Medicine, Department of Emergency Medicine, New York, New York

## Abstract

Point-of-care lung ultrasonography is an evidence-based application that may play a vital role in the care of critically ill pediatric patients. Lung ultrasonography has the advantage of being available at the patient’s bedside with results superior to chest radiography and comparable to chest computed tomography for most lung pathologies. It has a steep learning curve. It can be readily performed in both advanced healthcare systems and resource-scarce settings. The purpose of this review is to discuss the basic principles of lung ultrasonography and its applications in the evaluation and treatment of critically ill pediatric patients.

## BACKGROUND

Point of care lung ultrasonography has had a vital role in the care of critically ill patients for the past three decades. Currently, many pediatric emergency departments (ED) and intensive care units (ICU) worldwide employ it for patient care. International expert consensus and evidence-based recommendations support the use of lung ultrasound for evaluation and management of a variety of pulmonary pathologies in adult and pediatric patients.^1^ We present a review of applications of point of care lung ultrasound (US) in critically ill pediatric patients.

## INTRODUCTION

The formation of an image on an ultrasound machine involves generation of sound waves by piezoelectric crystals in the device’s transducer. Depending upon the acoustic impedance of the imaged structures, these sound waves are reflected, scattered, absorbed, or attenuated as they pass through different mediums of the body such as soft tissue, air, fluid, or bone.^2^ As air is a poor conductor of sound waves, the lungs were traditionally viewed as not suitable for ultrasound imaging. However, lung ultrasound is possible by interpretation of artifacts generated by the pleural line for aerated lungs and by direct visualization for many pathologies.^3^

## TECHNIQUE

- *Patient position:* Children are great candidates for lung US due to their thinner chest wall and smaller thoracic width compared with adults. Lung US can be performed with a child in any position (eg, sitting, supine, or in a parent’s arms) if appropriate images of bilateral anterior, lateral, and posterior lungs can be obtained. When available, warm gel should be used to increase patient comfort.
- *Probe:* A high-frequency linear probe (7.5–10 megahertz) is often used for lung US in pediatrics, as it offers high-resolution images of the pleural line and lung pathology.^4^ A lower frequency phased array or curvilinear probe may be used as well, depending on the patient’s body habitus.^1^
- *Probe orientation:* The probe can be placed longitudinally, perpendicular to the ribs, with the probe marker oriented toward the patient’s head. For a transverse approach, the probe marker should be placed transversely/obliquely, parallel to the intercostal spaces, with the probe marker oriented toward the patient’s right. The longitudinal approach allows visualization of the pleural line between two ribs, whereas the transverse/oblique approach enables increased visualization of pleura without rib interruption.^3^
- *Ultrasound Mode:* B-mode, or two-dimensional scanning, is used most commonly for lung US. M-mode, or motion mode, can be used for sonographic evaluation of the lung tissue, especially when evaluating for pneumothorax.
- *Scanning sites:* Lung US can only visualize pathology directly under the probe. Therefore, comprehensive scanning includes bilateral anterior, lateral, and posterior lung fields. However, the number of scanning sites can vary depending upon the clinical situation. In a critical care setting, for example, where the patient is supine, only the anterolateral chest may be accessible for scanning. Scanning of costophrenic angles is necessary to evaluate for fluid collection (e.g., pleural effusion or hemothorax).^3^

## LUNG SONOANATOMY FINIDNGS

### Pleural line

**Figure 1:**
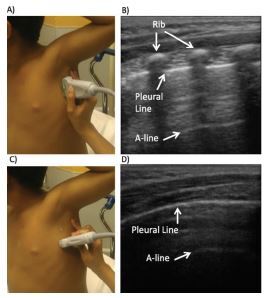
Normal lung ultrasound (US). A) Longitudinal probe positioning for lung US with B) corresponding longitudinal lung US image and bat sign. C) Transverse/oblique probe positioning for lung US with D) corresponding lung US image.

**Figure 2:**
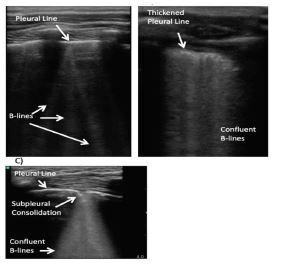
Alveolar interstitial syndrome. A) B-lines originating from the pleural line and extending to the bottom of the screen. B) “Waterfall” sign with confluent B-lines. C) Subpleural consolidation < 1 centimeter with confluent, trailing B-lines.

In a well aerated lung, the pleural line appears as a hyperechoic line with lung sliding. Lung sliding is the dynamic, horizontal, to-and-fro movement, or shimmering, of parietal pleura over visceral pleura with respiration. The pleural line is part of the bat sign in longitudinal view or can be visualized as an uninterrupted hyperechoic line in the transverse/oblique view (Figure 1). The bat sign consists of three hyperechoic areas: the two hyperechoic curved lines on the sides represent the upper and lower ribs with posterior acoustic shadowing, and the middle hyperechoic line between the ribs represents the pleural line.^2^ Clinically significant artifacts produced by pleural line are as follows:

1. *A-lines:* A-lines are static, horizontal hyperechoic artifacts arising from the reverberations produced between the pleural interface and the probe. The distance between each A-line is equivalent to the distance between the ultrasound probe and the pleural line.^5^ The A-line profile represents air in the alveolar spaces and is present in normal lungs, hyperinflated lungs, and pneumothorax.^3^ (Figure 1).
2. *B-lines:* B-lines or comet tail artifacts are discrete, hyperechoic, vertical artifacts arising from the pleural surface and extending to the bottom of the screen, obliterating A-lines at their intersection. The term lung rockets denote multiple B-lines in a lung scan. The number of B-lines in each intercostal space is related to the extent of fluid in the lungs: <3 B-lines is normal; 3–4 B-lines represent thickened interlobular septa; and >5 B-lines can represent severe interstitial disease^6^ (Figure 2A). Although there is no consensus on the physical basis of B-lines in the literature, multiple hypotheses suggest that the B-lines are produced by the acoustic properties of the pleura and a structural change in the geometry and connectivity of sub-pleural air spaces. It is hypothesized that transonic channels are formed by water accumulation in the pulmonary interstitium. These channels, along with the heterogenous collapse of terminal airspace, allow the propagation of US waves that would have been otherwise reflected in normal lungs. 7

## APPLICATIONS OF POINT-OF-CARE LUNG ULTRASOUND

Lung ultrasonography can be employed for various clinical conditions in the ED and ICU. It is readily available, easy to perform at the bedside, and has a significant advantage of reducing exposure to the ionizing radiation associated with chest radiograph (CXR) and computed tomography (CT). The following are common, evidence-based applications of lung US in children.

### Lung Consolidation

Lung US is superior to CXR and comparable to chest CT when employed in the evaluation of pneumonia in children. It has a steep learning curve but can be used in any clinical setting. Lung US may also differentiate between different etiologies of consolidation such as pneumonia, atelectasis, and pulmonary embolism.^1^

#### Scanning technique

In the evaluation for pneumonia, the sonographic technique begins with scanning the area of interest (eg, location of crackles on physical examination) and then progresses to scan the entire lung bilaterally, as needed. A comprehensive approach to lung scanning includes each intercostal space anteriorly in the mid-clavicular line, laterally in the mid-axillary line, and posteriorly in the paravertebral line.

A study of pediatric patients with pneumonia identified by lung US showed that most consolidations are posterior in location (47%), with 31% anterior and 23% lateral in location. In addition, more lung consolidations were identified in the transverse/oblique view than the longitudinal view (96% vs 86%).^8^ Therefore, complete lung scanning protocols with scanning in perpendicular planes are needed to minimize missing lung pathology.

#### Ultrasound findings

**Figure 3:**
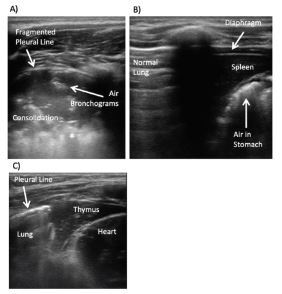
Pneumonia and potential pitfalls. A) Consolidation with fragmented pleural line, air bronchograms, and shred sign (*) consistent with pneumonia. B) Potential pitfalls of lung ultrasound for pneumonia include the spleen and air in the stomach below the diaphragm and C) thymus in the right upper anterior lung field adjacent to the heart in young children.

Lung with consolidation appears as a hypoechoic region deep to the pleural line due to the presence of fluid in the lung. Characteristic sonographic features of consolidation include hepatization, shred sign, air bronchograms, and focal/marginal B-lines. In addition, the pleural line may be hypoechoic or fragmented in the area of the consolidation (Figure 3).

1. *Hepatization:* A hypoechoic, homogenous, tissue-like appearance of the lung parenchyma, which corresponds to fluid and loss of aeration in the lung.
2. *Shred sign:* Refers to the irregular deep borders of the hypoechoic consolidation.^9^
3. *Air bronchograms:* Air in the bronchioles within the hepatized lung appear as hyperechoic structures within the hypoechoic, consolidated lung. As airways are patent in pneumonia, the air bronchograms move with breathing, which is known as *dynamic air bronchograms.* The movement of these air bronchograms differentiates a parenchymal disease such as pneumonia from non-patent airways, such as atelectasis.
4. Size of consolidation: A consolidation’s size may help differentiate between viral and bacterial etiologies of pneumonia. Viral pneumonia is usually associated with a small consolidation with a median diameter of 1.5 centimeters (cm), while bacterial causes of community-acquired pneumonia generally have consolidation size above 2 cm.^10^ A recent study found isolated sub-centimeter consolidations with no evidence of pneumonia on CXR or two-week follow-up. In contrast, sub-centimeter consolidations with large consolidations had the highest rates of definite radiographic pneumonia.^11^

The spleen and air in the stomach below the left diaphragm (Figure 3B) or the thymus in young children in the right upper anterior lung field (Figure 3C) may be mistaken for consolidation. Such errors can be avoided by keeping these potential pitfalls in mind and by paying attention to the location of the diaphragm while scanning the left lower chest.^12^ A meta-analysis of lung ultrasonography for the diagnosis of pneumonia has shown better sensitivity and comparable specificity to CXR.^13^ Lung US for diagnosis of pneumonia also has the ability to reduce the use of CXRs performed in the ED by 30–60%^14^ and decrease length of stay of patients in the ED by 48 minutes.^15^

### Alveolar Interstitial Syndrome

Alveolar interstitial syndrome (AIS) can be acute (e.g., viral infection, acute respiratory distress syndrome [ARDS], acute pulmonary edema, interstitial pneumonia), or chronic (e.g., pulmonary fibrosis). Point of care lung US is superior to conventional CXR for the diagnosis of interstitial lung disease and may lead to better patient outcomes. 1

#### Scanning Technique

While scanning for AIS, it is essential to do a comprehensive scan paying particular attention to the posterior lung bases, as the disease process typically starts there. In the case of limited time and limited patient mobility, scanning may be driven by patient pathology. For example, in a patient with cardiogenic pulmonary edema, scanning should include dependent zones of the lungs while an anterior scan should suffice for a critically ill patient with ARDS.^3^

#### Ultrasound Findings

AIS is characterized by pleural line abnormalities, the presence of multiple B-lines, and subpleural consolidations (Figure 2B and 2C).

1. *Pleural line abnormalities:* The pleural line can be normal (thin and regular) in cardiogenic causes of AIS, such as cardiogenic pulmonary edema. However, pulmonary causes of AIS (e.g., pulmonary fibrosis, ARDS, pneumonia) produce pleural line abnormalities such as increased thickness, fragmentation, irregularities, and absence of lung sliding due to adherence to exudates.^16^
2. *B-lines:* The presence of >3 B-lines per intercostal space is considered pathological (Figure 2B, 2C). Differences in patterns and uniformity of B-lines can differentiate between different etiologies of AIS. B-lines in a focal pattern can be seen in pathology limited to a specific lung region, such as pneumonia, atelectasis, pulmonary embolism, or neoplasm. In contrast, a more diffuse pattern of B-lines can be visualized in cardiogenic pulmonary edema, ARDS, and pulmonary fibrosis.^6^ Confluent B-lines, known as the waterfall sign (Figure 2B), may also occur.

### Bronchiolitis

Bronchiolitis is a common pediatric cause of AIS affecting children under 24 months of age, with infants 0–3 months of age having more severe disease. Lung US is more reliable than CXR for bronchiolitis and correlates well with clinical course.^17^

#### Ultrasound findings

The sonographic signs of bronchiolitis include small subpleural lung consolidations (<1–1.5 centimeters [cm]) (Figure 2C), presence of numerous compact or confluent B-lines defined as white lung, pleural line irregularity and, rarely, minimal pleural effusion or pneumothorax. In one study, lung US identified infants with bronchiolitis in need of supplementary oxygen with a specificity of 99% and sensitivity of 97%.^18^

### COVID-19

The coronavirus disease 2019 (COVID-19) pandemic has affected children worldwide and may have pulmonary features visualized on lung ultrasonography.

#### Ultrasound findings

Lung ultrasonography findings in children with COVID-19 include pleural line irregularities, B-lines (scattered and confluent), consolidations, and pleural effusions.^19^,^20^ In COVID-19, the waterfall sign of confluent B-lines can appear on and off, with normal lung parenchyma in between, representing an early phase of COVID-19 associated ARDS and corresponds with ground-glass opacities seen on chest CT.^21^ Newer portable ultrasound machines are easy to disinfect and allow for reduced patient movement between different hospital departments, which is especially important for infection control during the COVID-19 pandemic.^21^

### Trauma Applications

#### Near-drowning

Lung US has a potential role in evaluating drowning or near-drowning victims. Although there are no reported cases of its application in pediatric drowning victims, lung US has been shown to accurately diagnose the cause of acute respiratory failure in an adult patient with accidental near-drowning in seawater.^22^

#### Lung Contusion

Lung US findings that correlate with a contusion on chest CT include B-lines in a focal lung field,^23^ diffuse B-lines or AIS, and C-lines, which are defined as hypoechoic subpleural focal images with our without pleural line gap. In an adult study, lung US performed well compared to the gold standard when evaluating lung contusions. If AIS were considered a diagnostic criterion, lung US had a sensitivity of 95%, specificity of 96%, and accuracy of 95%. If C-lines were deemed diagnostic, the sensitivity and accuracy dropped to 19% and 66%, respectively, but the specificity increased to 100%.^24^

### Pleural Effusion

Lung US for diagnosis of pleural effusion is superior to CXR and as accurate as chest CT.

#### Scanning Technique

The lateral chest is scanned along the posterior axillary line to the diaphragm, which is the most dependent area of the chest in the supine patient.

#### Ultrasound Findings

**Figure 4:**
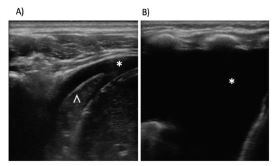
Pleural effusions. A) Small pleural effusion (*) at the costophrenic angle with consolidated lung due to pneumonia (^). B) Moderate-large pleural effusion (*) at the costophrenic angle.

Pleural effusion on US is seen as an anechoic or hypoechoic space between the chest wall and lung (Figure 4). Transudative fluid is generally anechoic, whereas internal echoes of the fluid may suggest exudative fluid or hemothorax.

Lung US can accurately quantify the effusion volume and indicate the appropriate location for thoracentesis. Ultrasound-guided chest tube placement in adults has a success rate of 97% and is currently a procedural standard of care in pediatrics.^25^

### Pneumothorax

Lung US has been shown to be more accurate than CXR for the diagnosis of pneumothorax and has similar accuracy to chest CT. In addition, lung US can differentiate between small and large pneumothoraces. Lung US for diagnosis of pneumothorax is easy to learn and has a steep learning curve.^1^

#### Scanning technique

As the air in a pneumothorax rises, the least dependent areas of the lung are scanned first. In the supine patient, the second intercostal space in the mid-clavicular line is the least dependent area. Lateral areas of the lung may be scanned to ensure that no pneumothorax is missed, especially if the patient is not supine or flat. Lung US has been shown to have a sensitivity of 98% and specificity of 99% for the diagnosis of pneumothorax in adults.^26^

#### Ultrasound Findings

**Figure 5:**
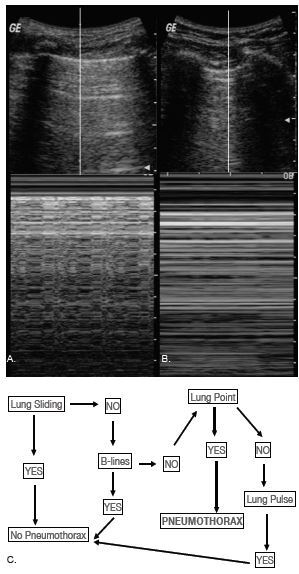
Pneumothorax. A) Normal aerated lung with the “seashore” sign on M-mode. B) Pneumothorax with the stratosphere/”barcode” sign on M-mode. C) Flow chart to evaluate for pneumothorax with lung ultrasound.^1^ *US*, ultrasound.

Ultrasonographic features of pneumothorax include an absence of lung sliding, presence of A-lines, presence of lung point, and absence of B-lines or lung pulse^1^ (Figure 5).

1. *Absence of Lung Sliding*: In pneumothorax, there is a static pleural line without the lung sliding seen in a normal, aerated lung. M-mode can be used to assess aeration of the lung below the pleural line by placing the M-mode scan line perpendicularly through the pleural line. For normal, aerated lung, M-mode shows the *seashore* sign, which is a noisy sonographic tracing below the pleural line. For pneumothorax, M-mode shows the *stratosphere* sign or *barcode* sign, which is as quiet below the pleural line as above.
2. *Lung Point:* The lung point denotes the edge of the pneumothorax and appears at the junction of pneumothorax with normal lung. In B-mode, lung sliding is opposed to the non-sliding pleural line, and in M-mode, the seashore sign is opposed to the stratosphere/barcode sign. The specificity of lung point for the diagnosis of pneumothorax is 100%.^27^ However, if the lung point is not visualized, it is generally a larger pneumothorax with a complete collapse of the lung. Therefore, identification of the lung point is not required for the diagnosis of pneumothorax.
3. *Absence of Lung Pulse:* The *lung pulse* is an artifact produced when cardiac pulsations are transmitted to the lung and chest wall.

An algorithm to evaluate for pneumothorax using these sonographic signs is shown in Figure 5C.^1^

### Ultrasound of the Diaphragm

Ultrasound of the diaphragm can be used to assess respiratory status. It has been shown to predict non-invasive ventilation failure in neonates^28^ and mechanical ventilation weaning outcomes for critically ill children.^29^ Recent evidence shows a correlation of diaphragm thickness and excursion with outcomes in patients with bronchiolitis and pneumonia in the ED.^30^,^31^ In addition, ultrasound of the diaphragm may have a role in triaging patients affected by COVID-19 ARDS. It may guide respiratory management decisions such as ventilation and the need for ICU-level care.^32^

#### Scanning Technique

**Figure 6:**
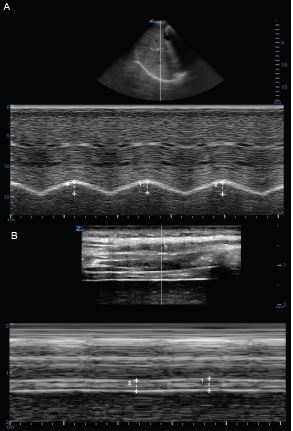
Diaphragmatic ultrasound. A) Diaphragmatic excursion on M-mode in subcostal view at the mid-clavicular line. B) Diaphragmatic thickness on M-mode at the mid-axillary line.

For diaphragmatic ultrasound, the phased array or curvilinear probe can be used to assess diaphragmatic excursion and thickness in the subcostal view, with the probe placed in the mid-clavicular line. The linear probe can be used to assess diaphragm thickness in the mid-axillary view (Figure 6).

- *Diaphragmatic excursion:* Diaphragmatic excursion evaluates the movement of the diaphragm with respiration. Diaphragmatic excursion with M-mode through the diaphragm appears as a sinusoidal wave; excursion is measured as the distance from the upward (inspiration) to downward (expiration) deflection, using the average of three breaths during baseline breathing.
- *Diaphragmatic thickness:* Diaphragmatic thickness is defined as the distance between the hyperechoic lines surrounding the diaphragm, which represent the diaphragmatic pleura and peritoneal membrane. The diaphragmatic thickness fraction (DTF) is determined by measuring the diaphragmatic thickness between inspiration when the diaphragm contracts and is at its thickest width (Tdi-Insp) and expiration when the diaphragm relaxes and is at its thinnest width (Tdi-exp) and is calculated with the formula: (Tdi-Insp – Tdi-exp)/Tdi-exp. The DTF has been shown to predict successful weaning of mechanically ventilated pediatric patients with a sensitivity of 82% and a specificity of 81%.^33^

### Placement of Endotracheal Tube

Placement of endotracheal tubes (ETT) is common in neonates, children, and adolescents presenting with respiratory failure. Chest radiography is the standard of care for confirmation of ETT placement. However, studies show >90% accuracy for confirmation of ETT placement with ultrasound.^34^

#### Scanning Technique

The scanning technique for confirmation of ETT placement involves the use of the following windows:

- Longitudinal mid-clavicular or mid-axillary intercostal view to confirm lung sliding and aerated lung bilaterally.
- Subcostal views assessing diaphragm motion bilaterally.

#### Ultrasound Findings

In the case of mainstem bronchus intubation, absence of lung sliding, non-aerated lung, and lack of diaphragmatic movement on the affected side would be seen. There may be a reversal of diaphragmatic movement when positive pressure is delivered to the stomach in esophageal intubation. When the patient is properly ventilated, diaphragmatic excursion will be visualized on M-mode as a sinusoidal wave (Figure 6A).

Diaphragmatic excursion for evaluation of proper ETT placement showed a sensitivity of 91% and specificity of 50%. Lung US has also been found to be quicker than conventional CXR (mean 19 vs 47 minutes, respectively) and comparable to capnography for confirmation of ETT placement.^34^

## LIMITATIONS

Limitations of lung ultrasonography include operator-dependent scanning skills, patient factors, and pathology factors. Although US is operator-dependent, point of care lung ultrasonography is a technique with a steep learning curve for many applications, novices have been shown to be able to perform lung US with high accuracy, and there is high interobserver agreement on image acquisition and image interpretation.^35^,^36^ Patient factors that may be limitations for bedside lung ultrasonography include obesity and subcutaneous emphysema. Pathology dependent limitations include etiologies that do not extend to the pleural surface. Therefore, it is important to note that centrally located consolidations, including perihilar or retrocardiac consolidations, that do not extend to the pleural line may be missed on lung US. However, more than 95% of pathological changes have a pleural component in both adults and pediatrics.^37^,^38^ Finally, most studies have a small sample size and compare lung ultrasound to CXR instead of lung CT, which is considered the gold standard in diagnosing respiratory disease. However, the comparison of lung US to lung CT would be unethical due to the risk of radiation exposure.^39^

## CONCLUSION

Point of care lung ultrasonography can facilitate early diagnosis of pulmonary pathology and, therefore, improved outcomes^39^ in critically ill children. Children are great candidates for lung US due to their thinner chest wall and smaller thoracic width. It has a significant advantage over chest radiograph and CT, including the ability to be performed by the clinician at the bedside and the absence of ionizing radiation exposure, and it has been shown to be more sensitive than CXR and comparable to chest CT in a vast number of pulmonary pathologies. Lung US is beneficial for the diagnosis and management of lung pathology in critically ill children in both well-resourced locales as well as in resource-scarce areas.^40^
